# Learning principles of evolution during a crisis: An exploratory analysis of student barriers one week and one month into the COVID‐19 pandemic

**DOI:** 10.1002/ece3.6741

**Published:** 2020-09-15

**Authors:** Emily Driessen, Abby Beatty, Alexis Stokes, Sara Wood, Cissy Ballen

**Affiliations:** ^1^ Department of Biology Auburn University Auburn AL USA

**Keywords:** biology, equitable practices, online learning, study habits, undergraduate students

## Abstract

The coronavirus disease (COVID‐19) outbreak forced an emergency transition to online classes across the world with little warning or instruction for faculty and students. The goal of this research was to document how this response impacted undergraduate students studying the principles of evolution in an introductory organismal biology class over time; specifically, how their study habits for exams differed (a) one week and (b) one month after a university's decision to transition to emergency remote instruction. We asked students about the extent to which COVID‐19 impacted their study habits, and we categorized students’ responses using open coding. We identified a number of consistent similarities—as well as dramatic differences—in their responses as the time away from campus increased. The report that follows is a summary of the documented barriers and recommendations based on literature concerning crises and equitable practices.

## Graphical abstract

The goal of this research was to document how the response to COVID‐19 impacted undergraduate students studying the principles of evolution in an introductory organismal biology class over time; specifically, how their study habits for exams differed (a) one week and (b) one month after a university's decision to transition to emergency remote instruction. The report that follows is a summary of the documented barriers and recommendations based on literature concerning crises and equitable practices.

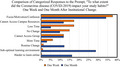

## 1 INTRODUCTION

In the last decade, college students have dealt with tragedies or disasters on national, local, and individual levels. These incidences have strong emotional and cognitive impacts on students—even those who are not directly affected (Honos‐Webb, Sunwolf, Hart, & Scalise, 2006). Examples include natural disasters such as Hurricane Maria, mass shootings (e.g. at the University of Texas, University of Iowa, and Virginia Tech; Post, 2016), local tragedies such as the murder of a fellow student (Mettler, 2020), and individual tragedies such as the sudden death of a family member. In response to the widespread prevalence of such tragedies, previous work documents campus actions that provide emotional support. For example, crisis response teams at some institutions provide support to anticipate or respond to tragedies (Asmussen & Creswell, 1995; Honos‐Webb et al., 2006). Other institutions have secured venues specifically for students and personnel to congregate and learn more about an incident and process grief (Hurst, 1999).

Although the response to these disasters and tragedies are well documented, the COVID‐19 pandemic and its implications for higher education presented unique challenges and research opportunities to inform future learning transitions. For example, the COVID‐19 outbreak resulted in an emergency transition to online classes. Learning in this context presented practical and complex obstacles—including difficulty accessing online course materials due to intermittent Internet access, processing the illness of a close family member, and preference for in‐person instruction. Teaching in this context also presented practical obstacles for faculty—online instruction requires a different skill set than in‐person instruction (Rohland‐Heinrich, 2016; Slovick, 2011). These skills take time and guidance to personally develop, neither of which were available for many instructors during the rapid transition. Additionally, a unique feature of the COVID‐19 response was the call for students to shelter in place for an indefinite period of time, cutting off contact to study groups and collaborative learning opportunities in class. Thus, for students enrolled in challenging, large science classes—particularly those that rewarded group work and participation—closure of the institution and self‐isolation resulted in a learning environment contradictory to the collaborative classroom settings to which students had grown to expect. Since the COVID‐19 pandemic erupted mid‐semester, instructors navigated multiple waves of challenges immediately after the closure of institutions through the end of the semester. This led us to investigate the following research question: How did the institutional transition to online instruction, a result of the COVID‐19 pandemic, affect student study habits over time?

To answer our question, we set out to document the challenges students faced as they studied for online examinations (a) one week and (b) one month after our institution's decision to transition to remote instruction. Our study took place in the context of a large, organismal biology class with an explicit focus on organismal evolution and classification. This course was taught at a southeast university in the United States (U.S.), so it is important to note the conclusions of this study may not be generalizable outside of this particular region or country. By using a repeated measures design, we captured collective snapshots of our students’ condition as they forcibly adapted to remote learning during this global emergency. We hypothesized that student challenges would markedly differ at these two time points, but some challenges would persist throughout the semester. For example, one week after the transition online we hypothesized students would report challenges related to the chaos of moving off campus and adjusting to online content delivery. This is because at that time, they had not grappled with learning content online for an extended time. In contrast, we expected that one month after the transition online, students may report persistent barriers they faced while adapting to the new norm of online coursework. By conducting exploratory studies that document the student experience, we contribute to a knowledge base that can inform institutional policy‐makers as well as instructors as we prepare for the future.

## 2 METHODS

### 2.1 Course background

We collected data in an Organismal Biology course from spring 2020, with a total enrollment of 415 students across two class sections. This class is the second in an introductory biology sequence taught at a research intensive, land‐grant university in the southeast region of the United States, with two of the paper's authors as the instructors of record (AB and CJB). The principle objective of the course is for students to develop an understanding of the evolution, classification, structure, and spectacular diversity of living organisms, focusing on plants and animals. This course is designed for those in biology‐related majors to prepare them for future coursework. In this course, students were assigned to small groups (ranging from 4 to 6 members) at the beginning of the semester and they sat and worked together for every class. It is a three‐credit class that includes two 75‐min class sessions each week and is offered every semester. The course initially met in a large auditorium, with seating for 270 students.

Each class period included lecture and group activities to reinforce the lecture material. For example, a typical class period may include some lecture, with 3–4 iClicker questions/group discussions every 15 min, as well as a 20‐min group activity. Students also took a group exam following individual examinations, allowing for students to confer with their group members over exam materials.

During the first ten weeks of the semester, students completed the first unit, including both the lessons and the first examination as well as the lesson material for the second examination. The students were then released for spring break with the plan to take the second examination in person upon returning from break. However, unpredictably, the university canceled in‐class meetings four days before students were to return from spring break due to the COVID‐19 pandemic. For that reason, class instruction became fully online four days after the cancelation of in‐class meetings, and instruction was to remain online for the rest of the semester (i.e., 7 weeks). Students then took their second examination 1 week after the transition online and took their third examination 1 month following the transition to the online format. Following the transition to online teaching, the instructors made all prerecorded “mini‐lectures” available, via Canvas, to students for the rest of the semester and required students complete a 10‐question “quarantine quiz” that corresponded to each class period. Of note, neither instructor had formal experience teaching online, but both had some experience with taking online classes as students.

### 2.2 Data collection

Immediately after students completed exams, both one week and one month after the transition online, we encouraged students to take a voluntary postexamination survey online via Qualtrics in exchange for a small amount of extra credit (awarded for clicking on the link to the survey). We selected an open‐ended survey question that could not be answered with a simple yes or no. Rather, we probed for elaboration concerning changes in student study habits: “to what extent did the Coronavirus disease (COVID‐2019) impact your study habits?” Our study offered an unusual opportunity to examine students’ responses after quickly transitioning from one type of instruction to another, rather than opting‐in to a particular mode of content delivery. In this repeated measures study, we measured the same variable(s) from the same population at two or more time points (i.e., one week and one month after the online transition).

### 2.3 Data coding and analysis

We downloaded survey responses one week following implementation. We found 330 students responded to both survey items (80% of students). For the first dataset, two of the authors (CJB and EPD) created categories through first‐ and second‐cycle analyses (Saldana, 2015) using open and thematic coding, and the following six codes emerged: (a) focus/motivation/confusion, (b) cannot access campus resources, (c) cannot access group members, (d) no change, (e) less time, and (f) more time (Table 1). The responses to the survey question of concern (i.e., to what extent did the Coronavirus (COVID‐19) impact your study habits?) were assigned as many codes as fit, meaning they could fit more than one code. Less than 5% of responses did not match any of the codes, and they were left uncoded. Two coders coded to consensus, meaning both coders agreed on code assignments for all responses. We calculated percentages for each code by dividing the total number of responses assigned for each code by the total number of student responses.

**Table 1:** Emergent themes in student open responses

Code	Definition: Students…	Example
Focus/motivation/confusion	…reported a variety of emotions, which in turn impacted productivity/learning	“Having to leave and not being able to have a quiet place to study at home living with a family of six took focus away from studying.”
Cannot access campus resources	…reported that physical study materials or access to the instructor or learning assistants were not available	“I enjoyed going to the library to study. I can no longer do that.”
Less time	…had less time to study and focus on their school work	“My children are also out of school, and I am balancing my time between ensuring that they are doing what they need to for classes and doing my own school work.”
No change	…were not impacted by the pandemic	“It did not impact my study habits.”
Cannot access group	…experienced fewer in‐person meetings with group members, with whom they were required to sit in class	“I was unable to study with friends and had no way of knowing if my knowledge had holes in it. Collaboration is key to my academic success.”
More time	…had more time to study and focus on their school work	“It actually helped because it gave me more time to chill and less time to travel.”
Routine change	….experienced a disruption in daily habits or structure	“I've become more of a procrastinator without a normal routine.”
Suboptimal learning	…experienced a less than ideal learning environment where they studied away from campus	“I am not used to being home and studying around my family. My family is very loud and I can never get any peace or quiet. I am not able to go to my usual designated study spots or even leave the house at all.”
Harder to learn online	…were challenged by online learning, compared to in‐person instruction	“COVID 19 made studying a little harder because online lectures are distracting for me.”
Easier to learn online	…preferred online learning, compared to in‐person instruction	“I found it easier to be able to understand the concepts more when having the lectures recorded and in front of me.”
Illness	…had family members, close friends, or they were ill	“COVID−19 impacted mine because I have been worried about my immunocompromised family member getting sick, and I think I'm already sick from going through the airports. I've been focused on helping my family, and I've had to be the one running the errands.”
Other	Responded in a way that was either vague or didn't address the impact COVID‐2019 had on their study habits.	“It has made me study a lot more.”

In response to the one month survey, we found 296 students responded to both survey items (71% of students). Four of the authors (EPD, AS, SW, and CJB) created an additional 5 codes to account for new emergent themes: (a) routine change, (b) suboptimal learning environment, (c) more difficult to learn online, (d) illness, and (e) easier to learn online. The same two coders coded to consensus. After the 1‐month data were coded, the two coders reanalyzed the 1‐week dataset with the additional 5 categories and coded to consensus again. Then, we calculated percentages for each code.

## 3 RESULTS AND DISCUSSION

**Figure 1:**
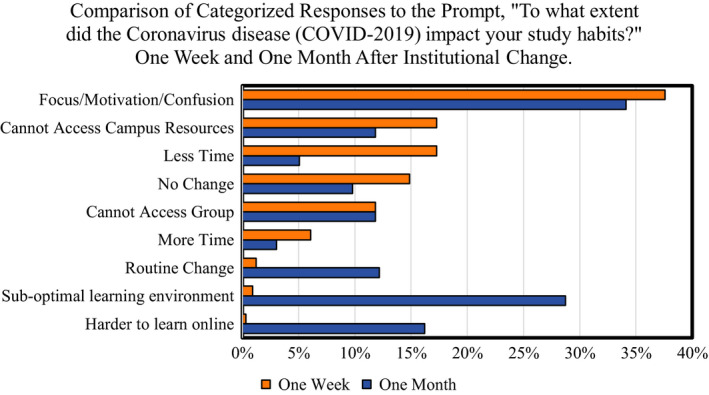
Student responses to the prompt, “To what extent did the Coronavirus disease impact your study habits?” one week (orange) and one month (blue) after the COVID‐19 transition online. All responses were assigned as many codes as relevant, meaning they could fit more than one code. Percentages were then calculated for each code by dividing the total number of responses assigned for each code by the total number of student responses. This figure displays only the six highest percentages for each dataset. Values indicate the percentage of students who reported each category as an impact of the institutional changes

Results consisted of one survey question answered at two time points: one week and one month after the university's transition to online instruction in response to the COVID‐19 pandemic. We received a total of 330 survey responses one week after the transition (80% of students) and 296 survey responses one month after the transition (71% of students). We asked “to what extent did the Coronavirus disease (COVID‐2019) impact your study habits?” and developed categories that encompassed most student responses (Table 1). The three most common student responses one week after transitioning online included focus/motivation/confusion (37.58% of responses), less time to study (17.27%), and not being able to access campus resources (17.27%); the three most common responses 1 month after transitioning included focus/motivation/confusion (34.12% of responses), a suboptimal learning environment (28.72%), and it is more difficult to learn online (16.22%) (Table 2, Figure 1).

**Table 2:**
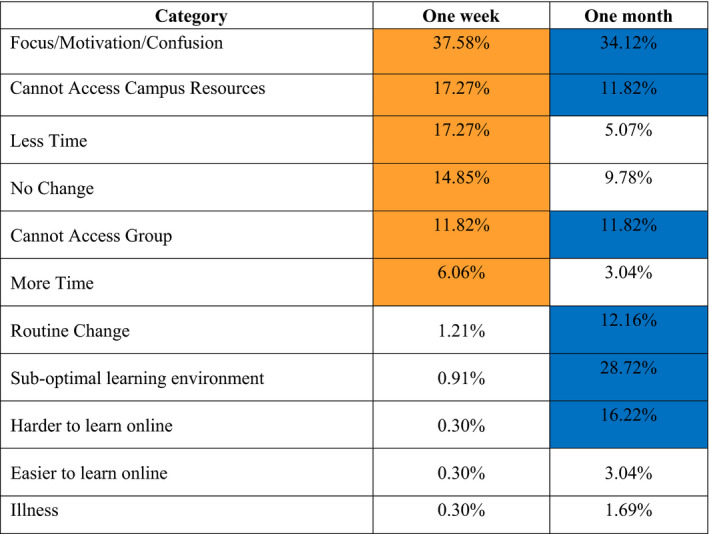
Comparison of categorized responses to the prompt, “To what extent did the Coronavirus disease (COVID‐19) impact your study habits?” at two time points: one week after and one month after the university switched to online‐only instruction in response to the COVID‐19 outbreak

Note

The six most common emergent themes for both time points are highlighted in orange (1‐week data) and blue (1‐month data). The percentages represent the number of students who mentioned the category as divided by the total number of student respondents.

A number of themes differed between student responses one week and one month after a switch to online instruction. For example, 1 week after the transition to online learning, students emphasized challenges related to the chaos of moving off campus that effected their access to campus resources as well as the amount of time they had to study (e.g., “It was harder to study because we had to travel home and pack up things from our dorms”) and their focus (e.g., “[The COVID‐19 pandemic] created a sense of panic and caused distraction from studying”) (Table 2; Figure 1). On the other hand, one month after the transition online, students emphasized (a) issues related directly to online instruction (e.g., “Harder to study because all of the material had to be learned online and by myself”); (b) a suboptimal learning environment (e.g., “I cannot focus at my house and I have three siblings and they interrupt me during my tests and I cannot focus”); or (c) the lack of structure in their daily routine (e.g., “it broke my class‐library‐work routine, and it has been more difficult to stay on top of my work without this kind of organized self‐discipline”). These differences made sense, because after one week students had just moved off campus and were adjusting to the online learning environment. After one month, the prospects of returning to campus were waning. At this time, students were sheltering in place and expected to interface with a virtual classroom—something many of them had never experienced in their education.

Though different patterns emerged when we compared student responses across the two time points, some themes dominated throughout the entire study period. One recurring theme from students was the negative impact of emotional factors on their study habits, such as anxiety, a sense of confusion, and lack of motivation (Table 2; Figure 1). While these were all placed in the same category during coding, the detailed responses put them in a more nuanced context; and differed 1 week or 1 month after transitioning online. For example, 1 week after transitioning online, students reported lacking motivation to study, with one student explaining that they were “less worried about this examination because there are a lot of more important things going on in my life right now.” It was common for students who cited a lack of motivation one month after the transition online to also describe distractions at home or the lack of a routine/structure. For example, “I have very little motivation to get things done at home since there is not really any structure.” This result highlights the persistent and potentially damaging impact that emotional factors have on student learning and well‐being during an extended crisis period.

Our results yield implications of this work that are twofold: exploratory studies can inform (a) institutional and (b) instructional policies in times of crisis.

First, institutions that seek feedback in the wake of COVID‐19 can develop an informed response in the case of future emergencies. For example, students in our sample reported COVID‐19 negatively impacted their emotional well‐being, which in turn affected their study habits, and this persisted over time. Under normal circumstances—those uncomplicated by a global pandemic—23.2% and 15.4% of American college students’ academic performances were negatively impacted by anxiety and depression, respectively (American College Health Association, 2016). To prepare for the additional emotional struggle experienced during a crisis, some institutions have developed emergency response teams to support or respond (Asmussen & Creswell, 1995; Honos‐Webb et al., 2006). At our institution, and presumably others, emotional support opportunities through the student counseling and psychological services closed for physical safety in response to the virus, even after instruction resumed. Thus, students were not able to obtain emotional or psychological support during the height of the crisis when it would have benefited the most students. Our results highlight the importance of this resource.

The second implication of this work is that exploratory studies can inform instructional practices in times of crisis. Based on personal communications with instructors, many experienced an uptick in student emails detailing logistical or emotional struggle while sheltering in place. However, these direct communications come from only a subset of students who are comfortable reaching out to an instructor (Miller & Pearson, 2013). We collected data from the majority of students in the class (i.e., between 70%–80% of students participated), decreasing the chance of a biased representation of student input. By formalizing data collection and using qualitative coding, where possible, instructors can better understand the most salient challenges for students and respond by adapting their course materials. In addition to aiding in evidence‐based instructional decisions, previous work also shows students respond positively when they perceive instructors care about their well‐being and exhibit warmth and respect for students (Dewsbury & Brame, 2019). Sharing anonymous student feedback from the survey with the class also has the potential to foster a sense of community (Phelan, 2012).

### 3.1 Other considerations and future directions

We are aware that instructors everywhere have been faced with countless examples of struggle through communications with students. This study offers insights into student experiences from two sections of one introductory biology course at a large southeastern university. While our survey was imperfect, the simple question about COVID impacts yielded meaningful insights. Similar work should be conducted in different geographic locations and at different institution types, where students might be facing different challenges. For example, the state in which the current research was conducted implemented a delayed stay‐at‐home order relative to other states and lifted the order relatively early. In contrast, California was the first state to transition online and ask its residents to shelter in place. We may expect students taking similar coursework to be differently impacted due to these circumstances, and therefore optimal instructor responses and institutional supports might also differ.

## 4 CONCLUSION

Our study exposed the tremendous strain COVID‐19 placed on students and their families. Teaching during this crisis confronted instructors with inequities that are undeniable, with differences in access to technology and levels of financial stability; students reported caring for siblings, if they had parents who were essential workers, or caring for their children who could not attend child care. Students themselves took on essential jobs to support themselves or their families. Locally, Alabama, the state in which our institution is located, experienced secondary weather emergencies that left students and their families without shelter. By offering this simple question to students, we gained a glimpse into their lives and observed stark differences based on socioeconomic status. These inequities underscore the importance of implementing equitable institutional policies and teaching practices to ensure that all students have the opportunity to excel, especially in a time of crisis. We hope this exploratory study can serve as a helpful resource for researchers and instructors who are interested in researching barriers or developing a response for their students.

## CONFLICT OF INTEREST

The authors of this article have no competing interests that would impact the integrity of this work.

## AUTHOR CONTRIBUTION

**Emily Paige Driessen:** Conceptualization (lead); Data curation (equal); Formal analysis (supporting); Methodology (equal); Writing‐original draft (lead); Writing‐review & editing (lead). **Abby Beatty:** Writing‐original draft (supporting); Writing‐review & editing (supporting). **Alexis Stokes:** Formal analysis (lead). **Sara Wood:** Formal analysis (lead). **Cissy Ballen:** Conceptualization (lead); Data curation (equal); Investigation (supporting); Methodology (supporting); Writing‐original draft (equal); Writing‐review & editing (supporting).

## Data Availability

Due to ongoing research with this dataset as well as IRB restrictions, data are not publicly available but are available upon request.
